# Ten-Year Monitoring of Bovine Mastitis-Causing Bacteria in Northern Italy and Evaluation of Antimicrobial Resistance in Raw Milk

**DOI:** 10.3390/microorganisms14010046

**Published:** 2025-12-25

**Authors:** Arianna Guaita, Franco Paterlini, Antonella Posante, Monica Boldini, Cinzia Rolfi, Paolo Daminelli

**Affiliations:** 1 National Reference Centre for Bovine Milk Quality, 25124 Brescia, Italy; paolo.daminelli@izsler.it; 2 Istituto Zooprofilattico Sperimentale Lombardia ed Emilia Romagna, 25024 Brescia, Italy; franco.paterlini@izsler.it (F.P.);

**Keywords:** ten-year trend, prevalence, mastitis-causing pathogens, antimicrobial resistance

## Abstract

Bovine mastitis is a multifactorial disease defined by the inflammation of the udder in cattle. It can be caused by different factors, but contagious or environmental pathogens play a major role in the onset of this disease. The main treatment for this condition is the administration of antibiotics, either parenterally or via the intramammary route. The samples were processed by the National Reference Centre for Bovine Milk Quality (CRNQLB) and bacteriologically examined by the IZSLER Primary Production Department (BS, Italy) over the period from 2015 to 2024. Moreover, this study presents the minimum inhibitory concentrations (MICs) obtained from all the bacterial pathogens isolated in the last three years of the study (2022–2024). This study aimed to describe the main frequencies recorded during the decade, in order to provide an enumeration of pathogens circulating in the IZSLER jurisdiction and to estimate trends in antimicrobial resistance, highlighting increases or decreases in observed resistance levels. Results show an increased prevalence of *Streptococcus uberis*, *Escherichia coli*, and *Enterococcus faecium*, with a decrease in *Prototheca*, yeasts, *Staphylococcus aureus*, and *Streptococcus agalactiae*. The general increase in antimicrobial resistance to trimethoprim needs to be highlighted to express the need for a targeted therapy based on accurate diagnosis to limit the spread of resistance in dairy farms.

## 1. Introduction

Bovine mastitis is one of the most widespread diseases of dairy animals. It is defined as an inflammation of the mammary tissue, caused by specific microorganisms, chemical irritation, or physical trauma [1]. Among the microorganisms involved in bovine mastitis, there are different classes of bacteria, yeasts, and unicellular algae, which enter the mammary gland through the udder sphincter [2]. Mastitis can be categorized based on clinical presentation (clinical/subclinical or chronic/acute) or according to the pathogens that play a major role in its development. In this case, bovine mastitis can be classified as contagious, caused by pathogenic bacteria such as *Staphylococcus aureus*, *Streptococcus agalactiae*, and the genus *Prorotheca* spp., or environmental, including members of the family *Enterobacteriaceae* [3]. Contagious pathogens are transmitted from infected to healthy cows, usually during milking, when tools are not properly sanitized between milkings. Environmental pathogens originate from the animal’s surroundings, including bedding, soil, manure, feces, and stagnant water [4]. The Automatic Milking System (AMS), a technology that implements the milking process and promotes animal welfare, must also be considered as a potential vector of transmission of mastitis-causing pathogens, despite the cleaning and disinfection procedures performed between milkings [5].

Bovine mastitis is a multifactorial disease with several risk factors, which makes it difficult to control. Nevertheless, effective management is crucial due to its economic and health impact, as it can lead to reduced milk production, changes in milk composition and quality, and, in severe cases, culling [6]. According to Regulation (EU) 2019/6, which governs the use of veterinary medicinal products, antibiotic administration is currently the only recognized effective treatment for mastitis [7]. However, conventional antibiotic use can promote the emergence of antimicrobial-resistant strains; in fact, excessive and often inappropriate use has contributed to the spread of multidrug-resistant microorganisms (AMR) [8]. These microorganisms have recently become a major public health concern due to their involvement in infections in both humans and food-producing animals, making AMR a global health challenge. To mitigate these risks, several regulatory and monitoring measures have been implemented. Among them, the prohibition of antibiotic use as growth promoters or for preventive purposes, introduced by Regulation (EU) 2019/6, has established stricter conditions for prescriptions [7]. In addition, Regulation (EC) No. 470/2009 sets maximum residue limits (MRLs) for pharmacologically active substances in foodstuffs of animal origin [9]. Complementing this regulatory framework, the ClassyFarm system, implemented in 2017, enables the monitoring of antimicrobial use at the farm level [10], taking into account the antimicrobial classification defined by the Antimicrobial Advice Ad Hoc Expert Group (AMEG) [11].

The antimicrobial classes most frequently used worldwide for the treatment of intramammary infections include β-lactams (penicillin and cephalosporins), aminoglycosides, lincosamides, and macrolides [12]. These antimicrobials are most commonly administered via the intramammary route; however, parenteral administration is also often employed for the treatment of clinical mastitis [13]. On farms, the conventional use of these antibiotics has readily led to the emergence of resistant strains. Nevertheless, recent studies have reported an alarming increase in resistance to newer antimicrobials, including piperacillin, ceftazidime, cefquinome, tigecycline, colistin, and vancomycin, highlighting the growing challenge of antimicrobial resistance in bovine mastitis [14].

As previously mentioned, the lack of proper attention to antimicrobial treatments can lead to serious health concerns. To mitigate the emergence of antimicrobial resistance (AMR) in bovine mastitis, accurate and rapid pathogen identification has acquired primary importance. Several diagnostic methods are employed to detect mastitis, each with advantages and limitations. The California mastitis test (CMT) is widely used as an on-farm screening tool, but it is based on the somatic cell count (SCC) as an indicator of inflammation [15]. It offers a rapid and low-cost means to identify potential mastitis cases, but does not provide information on the causative pathogen [16]. Although RT-PCR-based analyses are highly reliable, they require trained personnel and advanced laboratory equipment, which are not always available. Moreover, PCR can also detect DNA from non-viable microorganisms, potentially leading to an overestimation of infection [17]. For these reasons, the isolation of mastitis-causing agents through bacteriological culture remains the gold standard method [18].

Bacteriological culture analysis involves microscopic, culture-based, and biochemical investigations aimed at detecting and identifying mastitis-causing microorganisms. Isolation of the microorganisms allows their identification and is required for subsequent determination of the minimum inhibitory concentration (MIC), which determines the lowest concentration of an antimicrobial that inhibits bacterial growth. This analysis can be applied to a specific bacterial strain against multiple antimicrobials simultaneously. The resulting MIC values are then compared with the breakpoints reported in CLSI guidelines or by the European Committee on Antimicrobial Susceptibility Testing (EUCAST) [19,20].

The aim of this study is to provide a comprehensive overview of the prevalence of these pathogens in Northern Italy (Lombardy and Emilia Romagna) from January 2015 to December 2024, while evaluating resistance trends among the main pathogens identified in the past three years (January 2022–December 2024).

## 2. Materials and Methods

Bacteriological culture was performed on raw cow milk, collected under sterile conditions, with the initial streams from the teats discarded before sampling. All samples included in this study consisted of either fresh milk, stored at 5 °C ± 3 °C for a maximum of 48 h, or frozen milk samples obtained from individual quarters, individual cows, or bulk milk. These samples originated from self-monitoring analyses requested by farmers within the jurisdiction of the Primary Production Department of the Istituto Zooprofilattico Sperimentale della Lombardia e dell’Emilia Romagna (IZSLER, Brescia, Italy). Based on the owner’s request, samples were categorized into four groups: (1) bacteriological examination with Esculin Blood Agar medium (EBA) and Thallium Kristal Violette Toxin medium (T.K.T.); (2) detection of *Streptococcus agalactiae* with T.K.T.; (3) detection of *Staphylococcus aureus* with Baird Parker medium with RPF supplement (BP+RPF); and (4) determination of minimum inhibitory concentrations (MICs). All media used in this study were prepared in-house, following ISO 17025:2018 [21] procedures, and underwent the required functional testing prior to use. Table 1 summarizes the sample size included in the study. It should be noted that analysis of category 4 (MIC) included only samples collected from January 2022 onward, due to the adoption of the MIC method, which replaced the previously used Kirby–Bauer disk diffusion test.

Since the study considered mesophilic mastitis-causing agents, which grow at 37 °C, all inoculations were performed at 37 °C ± 2 °C for 24–48 h, based on the timing required for examination.

### 2.1. Plate Inoculation

When the contributor does not require the investigation of a specific pathogen, the bacteriological analysis is performed by inoculating 10 µL of milk both onto EBA and the selective differential medium T.K.T. The inoculations were carried out using 3 mm loop inoculators (10 µL) or calibrated micropipettes. All procedures were performed under a Bunsen flame to maintain sterility. Colonies of microorganisms seen at the end of incubation were identified based on morphological characteristics, growth patterns, microscopic appearance, and the results of confirmatory biochemical tests. Even after primary culture on EBA and T.K.T., morphological differences between colonies allowed a preliminary identification of some microorganisms. In case of uncertainty, specific biochemical identification tests were performed. In this study, the CAMP test was applied to confirm the presence of *Streptococcus agalactiae*, the tube coagulase test for *Staphylococcus aureus*, and a microscopic examination for *Prototheca* and yeasts [22]. Specifically, colonies of *Prototheca* and yeasts identified on EBA medium generally take on the coloration of the underlying medium but appear opaque. Microscopically, *Prototheca* colonies can be distinguished by a grayish opaque or dark blue appearance when methylene blue is used, whereas yeast colonies appear whitish-gray with a “rice-grain-like” morphology and can be either opaque or translucent depending on whether the surface is rough or smooth. Results were recorded as “Negative”, “Contaminated”, or “Positive”, with positive samples specifying the isolated microorganism and/or its genus [23]. “Contaminated” samples were defined as those containing more than two microorganisms on the same plate. If two pathogens could be clearly identified, each was isolated separately, giving priority to contagious microorganisms. If specific identification of a pathogen was not possible due to multiple contaminations, the sample was recorded as “Contaminated” (I).

The preparation of Blood Esculin Agar (EBA) medium in Table 2, the Thallium Kristal Violette Toxin (T.K.T.) medium in Table 3, and the Baird Parker with RPF supplement in Table 4 involves suspending the base ingredients in demineralized water at 50 °C and/or heating in a water bath until complete dissolution of reagents. The base medium is then distributed into containers of suitable volume, capped, and sterilized at 121 °C for 15 min. After sterilization, the medium is allowed to cool to the pouring temperature, and then the supplement is added. The mixture is homogenized and poured into Petri dishes. The sterile plates can be used after complete solidification or stored at 5 °C ± 3 °C for a maximum of 40 days for EBA and BP+RPF or 15 days for T.K.T. For these media, pH control (maintained at 7.3 ± 0.2 in EBA and 7.2 ± 0.2 in T.K.T. and BP+RPF), sterility testing, and quality control must be performed to ensure proper growth conditions.

The selective and differential Baird–Parker agar with modified Rabbit Plasma Fibrinogen supplement (BP+RPF) was used to detect coagulase-positive staphylococci in milk samples. As for EBA and T.K.T., 10 µL of raw cow milk was plated on Petri dishes and incubated at 37 °C ± 2 °C for 48 ± 6 h, followed by refrigeration at 4 °C ± 3 °C for at least 4 h. A positive result was visually identified by the presence of circular colonies with smooth surfaces and distinct margins, exhibiting a black coloration caused by the reduction of tellurite to tellurium by *Staphylococcus* spp. The simultaneous appearance of two white halos surrounding the colonies (a clear zone and an RPF halo) indicated the presence of coagulase-positive *Staphylococcus aureus*, as reported in ISO 6888-2:2021 “Microbiology of the food chain—Horizontal method for the enumeration of coagulase-positive staphylococci” [24].

### 2.2. Evaluation of the Minimum Inhibitory Concentration (MIC)

Post-isolation on EBA, antimicrobial resistance was evaluated in a total of 2838 field samples collected over the 2022–2024 period, corresponding to 30,265 analyses of pathogen–single antibiotic. The minimum inhibitory concentration (MIC) analysis was performed using ready-to-use 96-well microtiter plates (SensititreTM GramPositive MIC Plate—Thermo Scientific ^TM^, Waltham, MA, USA) [25], after incubation of isolated colonies in Mueller–Hinton broth (11 mL) (SensititreTM Cation Adjusted Mueller–Hinton Broth w/TES 100 × 5 mL, Thermo Scientific, Waltham, MA, USA) [26] for 18–24 h at 37 °C ± 2 °C. The plates, reporting 14 different antibiotics tested at the same time, were subsequently incubated for 18–24 h at 37 °C ± 2 °C and manually read by the operator. For isolates belonging to the genera *Streptococcus* and *Enterococcus*, the commercial Mueller–Hinton broth was supplemented with 280 µL of lysed horse red blood cells to ensure optimal growth. During this test, 14 different antibiotics at various concentrations were assessed, as reported in Table 5. The interpretation of results for clinical pathogens was based on the following reference sources: CLSI VET01S [19], 6th edition; EUCAST versions 11.0 and 13.0 [20], and CASFM 2020 [27]. Based on the literature and regulatory guidelines, the breakpoints (BPs) were defined to identify resistant pathogens.

### 2.3. Data Analysis

Data analysis was conducted using Microsoft Excel and R/RStudio 2024.04. [28]. The pattern of the analyses was based on relative frequency and prevalence studies. To achieve this, the results obtained from the reading of selective and differential media were processed through linear regression analysis, aimed at evaluating the significance of temporal trends and quantifying potential variations over time. Data were aggregated at a monthly level and derived from large sample sizes, which reduced short-term variability and limited the impact of autocorrelation. The analysis was based on the monthly relative frequency percentages calculated over the ten-year study period. The same approach was applied to the results obtained from EBA+T.K.T., T.K.T. alone, and BP+RPF media. The trends of individual bacterial genera were analyzed using a simple linear model based on the monthly percentage variation of isolates relative to the total number of positive samples for that month. From this, the linear coefficient and corresponding *p*-values (α = 0.05) were calculated. The same method was used to assess the isolation trends of individual bacterial species.

To evaluate the time trend of overall antimicrobial resistance in the period 2022–2024 and the resistance pattern of the most frequently isolated pathogens, a generalized linear model (GLM) with binomial distribution was applied, as resistance outcomes were expressed as proportions of resistant isolates over time. Each isolate was classified as resistant (R), susceptible (S), or intermediate (I) according to the MIC ratio values obtained.

This allowed the calculation, for each antibiotic, of the proportion of resistant strains, defined as the ratio between resistant samples (nR) and the total number of tests performed within the same period (nTOT), expressed as pR = nR/nTOT.

Variations in resistance observed among individual bacterial isolates were analyzed by establishing bacteria–antibiotic relationships and considering the monthly relative frequencies of resistance. The function of the logistic regression model used was the following:

$$logit\;\left(pR\right)=\;\beta_{0}+\beta_{1}\;\times \;time$$

where *pR* represents the probability of resistance, $\beta_{0}$ the model intercept, $\beta_{1}$ the regression coefficient describing the temporal trend, and “*time*” the monthly time variable. The statistical significance was set at α = 0.05.

Linear regression models and generalized linear models were used to evaluate temporal trends, as the primary objective was to assess long-term directional changes. Data were aggregated at the monthly level and derived from large sample sizes, which reduced short-term variability and limited the impact of autocorrelation.

## 3. Results

From an initial analysis based on relative frequency percentage, reported in Table 6, a significant decrease was observed in the proportion of negative samples (*p*-value 2.58 × 10^−5^) with a linear coefficient of −0.006 (daily average decrease corresponding to an estimated annual decrease of −2.06%/year). Conversely, positive samples showed a significant increase (*p*-value 5.99 × 10^−6^, linear coefficient 0.006), corresponding to an annual average increase of 2.05%, while no significant variation was detected for contaminated samples (coefficient 4.2 × 10^−5^, *p*-value 0.918).

**Table 6:** Annual relative frequency percentages of contaminated (I), negative (N), and positive (P) samples obtained from EBA and T.K.T.

Year	EBA+T.K.T.		
	I	N	P
2015	11.1	68.5	20.4
2016	15.1	55.5	29.4
2017	15.5	61.3	23.1
2018	20.9	51.4	27.7
2019	18.8	51.6	39.6
2020	13.1	55.8	31.1
2021	15.0	57.5	27.5
2022	15.4	57.7	27.0
2023	18.0	51.8	30.3
2024	18.3	48.6	33.2

From the evaluation of the results obtained with the inoculation of milk samples on T.K.T. medium, processed as a unique medium when the owner requests the selective isolation of *Streptococcus agalactiae*, it was observed that, despite a slight decrease in positive samples and a corresponding increase in negative ones (*p*-value 0.351 and 0.542, linear coefficients −0.0005 and +0.0003), no statistically significant trends in relative frequency percentages could be confirmed, as reported in Table 7. On the contrary, a significant increase in contaminated samples was detected with a *p*-value of 0.031.

Analyses performed on the selective differential medium BP+RPF used to isolate *Staphylococcus aureus*, show that the relative percentage frequencies (reported in Table 8) exhibited a significant increase in negative samples over the decade (*p*-value = 1.07 × 10^−9^; linear coefficient = 0.271). Linear regression analysis showed that the percentage of negative samples increased while that of positive ones decreased with similar statistical significance (*p*-value = 9.16 × 10^−10^ and linear coefficient = 0.273). As for contaminated samples, although the trend appeared slightly decreasing, no significant variation could be established (*p*-value = 0.384).

**Table 8:** Annual relative frequency percentages of contaminated (I), negative (N), and positive (P) samples obtained from BP+RPF.

Year	BP+RPF		
	I	N	P
2015	2.22	87.1	10.7
2016	2.04	85.7	12.2
2017	1.65	87.6	10.7
2018	2.40	84.0	13.6
2019	1.92	88.4	9.66
2020	1.94	91.3	6.73
2021	1.16	90.0	8.89
2022	0.97	94.1	4.96
2023	1.14	94.9	4.02
2024	2.78	92.2	5.21

### 3.1. Study of the Prevalence of Positive Samples on Blood Esculin Agar (EBA) and Thallium Kristal Violette Toxin (T.K.T.) Media with Ten-Year Trend Analysis

Furthermore, an analysis was conducted to assess the prevalence of bacterial genera and their trends over the study period. Among the 104820.00 samples analyzed on EBA and T.K.T. media, 29831.00 were positive.

The identification of bacterial genera and species was initially performed based on colony morphology, growth characteristics on selective and differential media, and microscopic examination. Confirmatory biochemical tests were applied when necessary: the CAMP test for *Streptococcus agalactiae*, the tube coagulase test for *Staphylococcus aureus*, and microscopic staining for *Prototheca* and yeasts. This approach allowed for presumptive identification, which was further confirmed for individual isolates where uncertainty arose.

As reported in Table 9, the most frequently detected genera were *Staphylococcus* (23.90%) and *Streptococcus* (20.51%). These were followed by the *Enterobacteriaceae* family (12.98%) and the genus *Enterococcus* (10.85%).

In addition, the most frequently isolated pathogens are reported in Table 10. For statistical analysis, only positive samples for which a definitive species-level identification was obtained were included in species-level trend calculations. Positive samples with ambiguous or multiple contaminating colonies that could not be resolved were recorded as “Contaminated” and excluded from species-specific analyses and from positive samples if not included in genus-level analyses.

Among the predominant species, *Streptococcus uberis* (13.66%) and *Escherichia coli* (12.23%) were the most frequently isolated. This analysis excluded all the positive results for which bacterial isolation did not lead to a definitive identification of the species causing the mastitis.

When assessing the temporal trends of pathogens, a significant increase (*p*-value 7.8 × 10^−6^) was observed only for the genus *Streptococcus*, which increased from 10.58% in 2015 to 28.78%, corresponding to a percentage change (Δ%) of +18.20%.

Significant increases were also recorded for isolates belonging to the family *Enterobacteriaceae* and the genera *Staphylococcus*, *Serratia,* and *Trueperella.* Conversely, a decrease was observed for *Enterococcus*, *Prototheca,* and yeasts, as shown in Table 11. Although *Proteus*, *Cirotrobacter*, and *Pseudomonas* displayed a decreasing trend, no statistical significance was detected over the ten-year period.

Out of the 29831.00 positive samples detected, 16581.00 were identified at the species level. Table 12 reports the annual variations in relative frequencies of each isolated pathogen, along with the analysis of their respective ten-year linear trends. A significant increase was observed in the isolation of *Streptococcus uberis* (*p*-value 1.67 × 10^−8^), *Escherichia coli* (*p*-value 0.0478), and *Enterococcus faecium* (*p*-value 0.0292). Conversely, a significant reduction was recorded for *Enterococcus faecalis* (*p*-value 1.23 × 10^−9^), *Streptococcus agalactiae* (*p*-value 0.0013), and *Staphylococcus aureus* (*p*-value 0.0019). No significant variations were detected for *Staphylococcus haemolyticus*, *Tueperella pyogenes,* and *Citrobacter koseri.*

### 3.2. Analysis of Antimicrobial Resistance Trends in Isolated Bacteria

Over the three-year period (2022–2024), based on 30.265 MIC values, the proportion of samples showing resistance to at least one antibiotic showed a decreasing trend (*p*-value = 0.0056), corresponding to a 13.5% reduction in the probability of detecting resistance. When assessing the probability of resistance over time for each bacteria–antibiotic pair, the model identified nine combinations whose variation over time was statistically significant, as reported in Table 13.

## 4. Discussion

As of 2025, bovine mastitis represents one of the major challenges in dairy farming, not only because of its considerable impact on the dairy economy but also due to its relevance within the One Health framework. The multifactorial etiology of mastitis requires an integrated and long-term approach, as effective control strategies must simultaneously address animal management, environmental conditions, pathogen circulation, and antimicrobial use. This complexity explains why, despite advances in herd management and diagnostic practices, mastitis continues to represent a persistent concern for dairy production systems [29].

**Figure 1:**
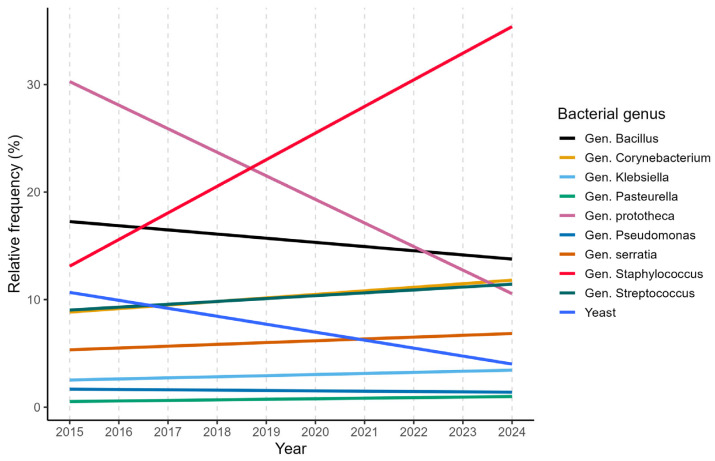
Linear annual trends of the 10 most frequent bacterial genera.

The main pathogens associated with bovine mastitis detected between 2015 and 2024 in Lombardy and Emilia Romagna belonged to the genera *Staphylococcus*, *Streptococcus*, *Enterobacteriaceae*, and *Enterococcus*, in agreement with the current epidemiological patterns of bovine mastitis reported in the literature [6,30,31] and shown in Figure 1.

This distribution reflects the coexistence of both contagious and environmental transmission routes within dairy herds. Among all positive field samples, specific pathogens of note are *Streptococcus uberis*, *Escherichia coli*, *Enterococcus faecalis*, *Enterococcus faecium*, as well as *Staphylococcus aureus* and *Streptococcus agalactiae*, which are already identified as the major mastitis-causing pathogens found in dairy herds [3,32]. The central role of these microorganisms in mastitis epidemiology highlights the need for control strategies, including rapid and accurate diagnostic procedures.

The bacteriological examination of milk represents the gold-standard analytical technique for the diagnosis of bovine mastitis. This method consists of a set of laboratory procedures aimed at isolating and identifying the microorganism responsible for the pathological condition [33]. However, the observed increase in contaminated samples over time on T.K.T. medium suggests that procedural factors occurring before the bacteriological examination may compromise analytical outcomes. This trend may reflect increased submission of samples from subclinical cases, higher awareness and surveillance activity, as well as persistent challenges in ensuring optimal sampling practices at the farm level.

A similar pattern was also observed in analyses performed on EBA+T.K.T. and BP+RPF media, although in these cases the variations were not statistically significant. Regarding positive and negative results obtained from EBA+T.K.T., a marked increase in positive samples was observed, accompanied by a proportional decrease in negative samples. This may reflect an increase both in surveillance intensity and in disease occurrence. Encouragingly, analyses specifically targeting *Streptococcus agalactiae* on T.K.T. showed a stable isolation frequency over the ten-year period, while those performed on BP+RPF for *Staphylococcus aureus* demonstrated a significant reduction in positive results. These trends strongly suggest the effectiveness of the preventive and control measures implemented to limit the spread of major contagious mastitis pathogens, as previously reported in the literature [7,34,35].

**Figure 2:**
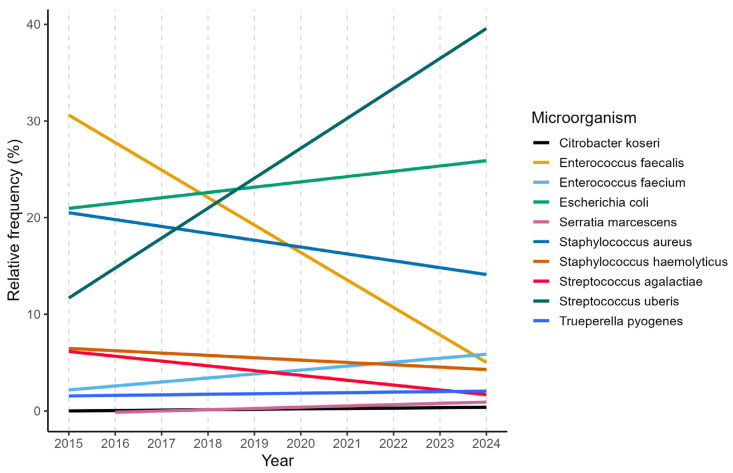
Linear annual trends of the 10 most frequent mastitis pathogens.

Following the assessment of overall diagnostic outcomes, the focus shifted to individual genera and species, revealing distinct and contrasting epidemiological patterns. An increase in the isolation frequency of several bacterial genera, particularly *Streptococcus*, *Staphylococcus,* and members of the *Enterobacteriaceae* family, was observed over the study period, in agreement with previous findings by Snulski et al. [36]. In particular, a significant increase was observed for *Streptococcus uberis*, *Escherichia coli* and *Enterococcus faecium*, as reported in Figure 2.

Their trend is known to be strongly influenced by housing conditions, bedding management, and overall farm hygiene. In contrast, a consistent decline was observed for traditionally contagious pathogens, including *Streptococcus agalactiae* and *Staphylococcus aureus*, as well as for organisms such as *Prototheca*, yeasts, and *Enterococcus faecalis.* These findings further support the sustained effectiveness of control strategies aimed at reducing cow-to-cow transmission, while highlighting the growing importance of environmental sources of infection.

**Figure 3:**
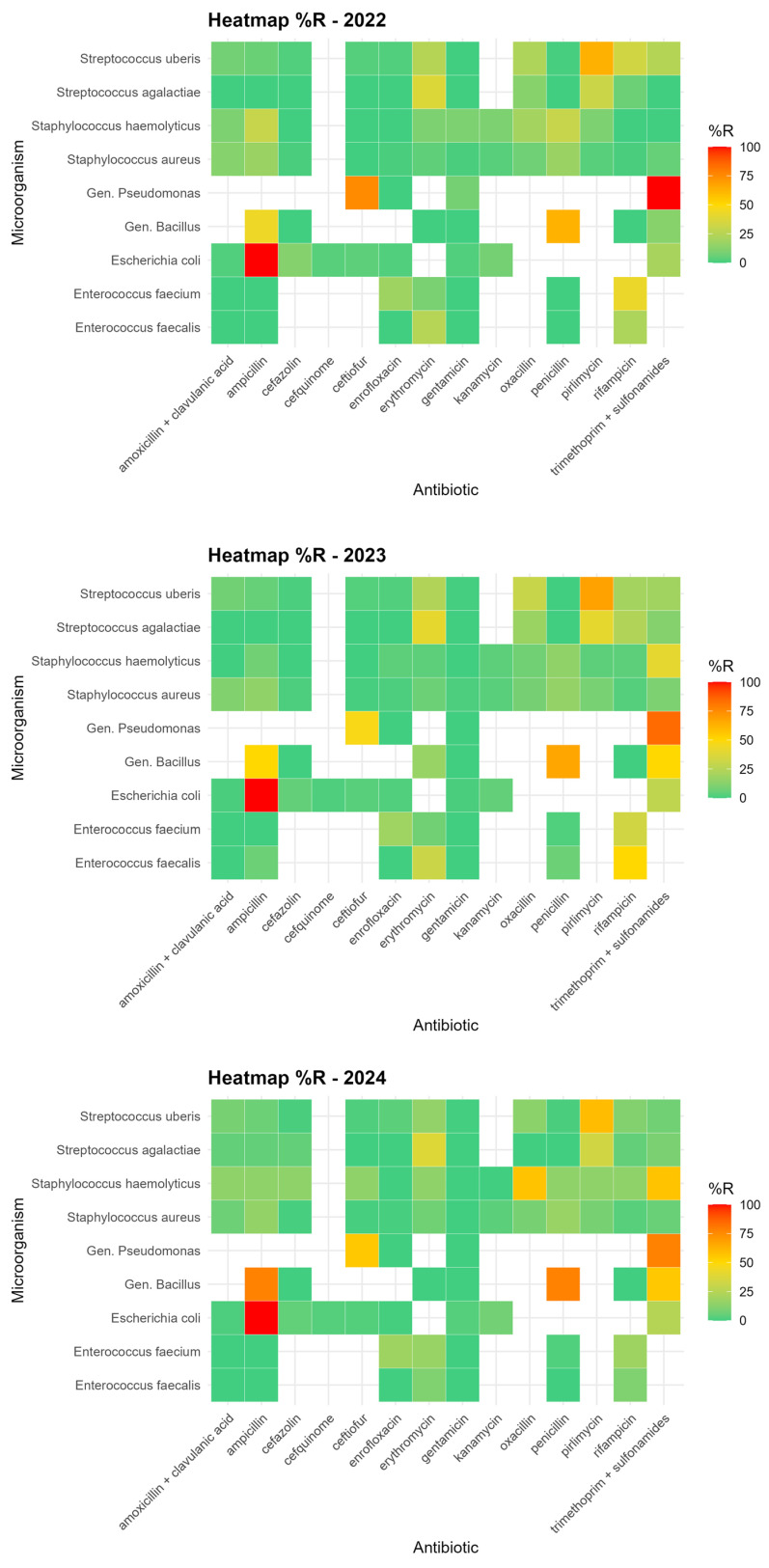
Heatmap showing the relative percentage frequency of microorganism–antibiotic combinations.

Antimicrobial resistance was evaluated using MIC-based antibiograms performed on commercial microtiter plates containing a broad panel of antibiotics commonly employed in bovine mastitis therapy. The tested compounds covered multiple antimicrobial classes, including β-lactams, aminoglycosides, macrolides, fluoroquinolones, lincosamides, and rifamycins, providing a comprehensive overview of resistance patterns among mastitis-associated pathogens circulating in the study area. Overall, resistance to several commonly used antibiotics was detected among mastitis-associated pathogens; however, high resistance levels were generally limited to specific pathogen–drug combinations or to a small number of isolates. Our analysis revealed that the antibiotics showing the most widespread resistance overall were pirlimycin, trimethoprim-sulfonamide, erythromycin, and ampicillin. However, with the exception of *Escherichia coli* (100% resistance to ampicillin), as already reported in the bibliography [37], and *Pseudomonas* spp. (87.5% resistance to trimethoprim-sulfonamide), high resistance rates were generally sporadic or limited to a few isolates, as reported in Figure 3.

Taken together, these findings suggest that there is no evidence of widespread, severe antimicrobial resistance in the IZSLER territory, although some pathogen–drug combinations exhibit localized critical patterns that persist and warrant continued attention.

The temporal analysis of antimicrobial resistance revealed an overall decreasing trend in the probability of detecting resistant isolates during the most recent years of the study period. This finding suggests a progressive improvement in antimicrobial stewardship practices within the territory, likely supported by increased use of diagnostic-guided therapy and heightened awareness of prudent antimicrobial use. Nevertheless, the modest proportion of variability explained by temporal factors alone indicates that resistance dynamics remain complex and are influenced by multiple interacting elements beyond time, including pathogen ecology, farm management practices, and antimicrobial usage patterns. Despite the overall favorable trend, certain resistance patterns remain concerning. In particular, increasing resistance to trimethoprim + sulfonamide observed in *Bacillus* spp. and *Staphylococcus haemolyticus* highlights the ability of some microorganisms to persist within herds despite sanitation, animal welfare, and mastitis control measures implemented in recent years [38]. These findings emphasize the need for continuous surveillance to promptly detect emerging resistance traits.

This study has some limitations that should be acknowledged. Although the analysis was based on samples routinely submitted to the CRNQLB, which reflect real-world diagnostic activity across the territory, the absence of a structured sampling framework limits the ability to directly infer high-level prevalence or establish causal relationships. In addition, detailed farm metadata (e.g., housing system, herd size, and antimicrobial usage) were not consistently available, limiting the ability to directly link epidemiological trends to specific management practices. Nevertheless, the results indicate that herd management strategies implemented in recent years have undoubtedly led to substantial improvements, particularly reflected in the reduced prevalence of major contagious mastitis pathogens such as *Streptococcus agalactiae* and *Staphylococcus aureus*. However, the persistence of environmental pathogens and selected resistance patterns suggests that these strategies must be further strengthened and consistently maintained over time to effectively limit both mastitis occurrence and associated antimicrobial resistance [31].

Proper milk sampling and accurate pathogen identification remain essential components of effective mastitis control. When preventive measures fail to fully contain infection spread, antimicrobial therapy should always be guided by etiological diagnosis and by the identification of herd-specific risk factors, in order to reduce the risk of infection and limit the emergence and dissemination of antimicrobial resistance [37,39,40].

## 5. Conclusions

Antimicrobial resistance remains a relevant concern in dairy farming; however, the results of this study indicate that the control strategies currently implemented in the study area are overall effective. These findings support the importance of maintaining high standards of on-farm hygiene and ensuring adequately trained farm staff capable of early detection of udder or milk alterations, which are essential components of mastitis prevention and control. From a practical perspective, the adoption of regular routine analyses supported by accurate and standardized diagnostic methods enables timely and targeted therapeutic interventions, thereby improving animal health, productivity, and antimicrobial stewardship. At the regional level, continuous microbiological and antimicrobial resistance surveillance represents a valuable tool to monitor evolving epidemiological trends, guide evidence-based decision-making, and support integrated One Health strategies aimed at safeguarding both animal and public health.

## Figures and Tables

**Table 1:** Number of samples included in the study.

Year	Method Employed			
	1.	2.	3.	4.
2015	12.985	20.131	26.154	-
2016	8.209	25.251	18.064	-
2017	8.603	19.486	13.745	-
2018	8.314	13.852	12.878	-
2019	7.641	8.392	9.679	-
2020	10.802	8.702	10.437	-
2021	13.319	11.705	12.679	-
2022	12.967	74.836	10.553	7.469
2023	12.335	4.204	9.334	10.586
2024	9.645	3.986	11.401	12.210
TOT	104.820	120.545	134.924	30.265

Method 1: EBA+T.K.T.; Method 2: T.K.T.; Method 3: BP+RPF; Method 4: MIC.

**Table 2:** Preparation of Blood Esculin Agar (EBA) medium.

Base Medium EBA	Concentration
Meat extract	10.0 g
Peptone	10.0 g
Sodium chloride (NaCl)	5.0 g
Esculin	1.0 g
Ferric citrate	100.0 mg
Agar	15.0 g
Demineralized water	1000.0 mL
Sterile defibrinated bovine blood supplement	70.0 mL

**Table 3:** Preparation of Thallium Kristal Violette Toxin (T.K.T.) medium.

Base Medium T.K.T.	Concentration
Meat extract	10.0 g
Peptone	10.0 g
Sodium chloride (NaCl)	5.0 g
Esculin	1.0 g
Ferric citrate	100.0 mg
Thallium acetate [TI(OCOCH_3_)]	334.0 mg
Agar	15.0 g
Crystal violet	1.8 mL
Demineralized water	1000.0 mL
Sterile defibrinated bovine blood supplement	70.0 mL

Staphylococcal beta-hemolysin added in variable volumes according to titration results (supplement).

**Table 4:** Preparation of Baird Parker (BP) medium + Rabbit Plasma Fibrinogen (RPF) supplement.

Base Medium BP	Concentration
Meat extract	5.0 g
Tryptone	10.0 g
Yeast extract	1.0 g
Sodium pyruvate (C_3_H_3_NaO_3_)	10.0 g
Glycine (C_2_H_5_NO_2_)	12.0 mg
Lithium chloride (LiCl)	5.0 mg
Agar	15.0 g
Demineralized water	1000.0 mL
RPF Supplement	
Bovine fibrinogen	5.0 g
Rabbit plasma with EDTA	10.0 g
Trypsin inhibitor	1.0 g
Water solution with 1% of potassium tellurite (K_2_TeO_3_)	10.0 g

**Table 5:** List of antibiotics tested for the detection of antimicrobial resistance in milk samples suspected of mastitis.

**Antibiotic**	**Class**	**Lowest µg/mL**	**Higher µg/mL**
Amoxicillin + Clavulanic acid	β-lactam	0.125	16
Ampicillin	β-lactam	0.03125	16
Penicillin	β-lactam	0.03125	16
Oxacillin	β-lactam	0.125	4
Cefazolin	Cephalosporin	0.125	8
Ceftiofur	Cephalosporin	0.125	32
Cefquinome	Cephalosporin	0.125	8
Enrofloxacin	Fluoroquinolone	0.125	4
Erythromycin	Macrolide	0.125	8
Gentamicin	Aminoglycoside	2/250	32/500
Kanamycin	Aminoglycoside	4	32
Trimethoprim + Sulfonamides	Dihydrofolate	0.125	4
Pirlimycin	Lincosamides	2	4
Rifampicin	Rifamycin	0.0625	2

It should be noted that gentamicin was tested at both low (2–32 µg/mL) and high (250–500 µg/mL) concentration ranges, in order to assess bacterial susceptibility across different dosing levels.

**Table 7:** Annual relative frequency percentages of contaminated (I), negative (N), and positive (P) samples obtained from T.K.T. only.

Year	T.K.T.		
	I	N	P
2015	1.15	90.8	8.01
2016	2.40	84.6	13.0
2017	1.40	88.0	10.6
2018	3.49	86.5	10.0
2019	2.41	88.5	9.06
2020	2.59	82.7	14.7
2021	3.31	89.1	7.58
2022	4.55	85.5	9.97
2023	0.73	88.7	10.8
2024	2.85	90.3	7.83

**Table 9:** Prevalence of bacterial genera isolated from bacteriological analysis with EBA and T.K.T. (2015–2024).

Genus	Prevalence% 2015–2024
Staphylococcus	23.90
Streptococcus	20.51
*Enterobacteriaceae* (Family)	12.98
Enterococcus	10.85
Prototheca	9.25
Bacillus	7.26
Corynebacterium	5.06
Yeast	3.38
Serratia	3.03
Klebsiella	1.38
Trueperella	0.93
Pseudomonas	0.73
Pasteurella	0.35
Citrobacter	0.15
Proteus	0.12
Aeromonas	0.05
Nocardia	0.03
Acinetobacter	0.02
Salmonella	0.01
Moraxella	0.00

**Table 10:** Prevalence of individual pathogens isolated from bacteriological analysis on EBA and T.K.T. (2015–2024).

Microorganisms	Prevalence% 2015–2024
*Streptococcus uberis*	13.66
*Escherichia coli*	12.23
*Staphylococcus aureus*	8.72
*Enterococcus faecalis*	8.69
*Staphylococcus haemolyticus*	2.75
*Enteroccoccus faecium*	2.16
*Streptococcus agalactiae*	1.87
*Trueperella pyogenes*	0.93
*Serratia marcescens*	0.20
*Streptococcus canis*	0.17
*Citrobacter koseri*	0.09
*Enterobacter cloacae*	0.04

**Table 11:** Statistical significance of ten-year trends in bacteriological genera isolated from field milk samples suspected of mastitis and submitted to IZSLER.

Genus	2015%	2024%	Δ%	Linear Trend (Coeff.)	*p*-Value
Streptococcus	10.58	28.78	18.20	0.0033	7.8 × 10^−6^
Enterococcus	19.85	7.78	−12.07	−0.0033	5.88 × 10^−8^
Prototheca	16.21	4.56	−11.65	−0.0026	3.84 × 10^−5^
*Enterobacteriaceae*	9.97	17.84	7.87	0.0009	0.0253
Yeast	8.42	3.06	−5.36	−0.0011	1.14 × 10^−5^
Bacillus	6.94	4.85	−2.09	−0.0002	0.6518
Staphylococcus	18.00	20.05	2.05	0.0016	0.0182
Serratia	2.89	4.19	1.30	0.0003	0.0111
Proteus	1.18	0.53	−0.66	−0.0002	0.0673
Klebsiella	1.52	2.18	0.65	5.4 × 10^−5^	0.6261
Citrobacter	1.08	0.58	−0.51	−7.78 × 10^−5^	0.1937
Trueperella	1.09	1.53	0.44	1.65 × 10^−5^	0.8542
Corynebacterium	3.60	4.02	0.43	0.00054	0.0429
Pseudomonas	0.97	0.63	−0.34	−5.75 × 10^−5^	0.3695
Pasteurella	0.89	0.57	−0.32	−7.22 × 10^−5^	0.2253
Nocardia	0.61	0.34	−0.27	−1 × 10^−4^	0.4149

**Table 12:** Statistical significance of ten-year trends in bacteriological microorganisms isolated from field milk samples suspected of mastitis and submitted to IZSLER.

Microorganism	2015%	2024%	Δ%	Linear Trend (Coeff.)	*p*-Value
*Streptococcus uberis*	4.33	22.41	18.08	0.0039	1.67 × 10^−8^
*Enterococcus faecalis*	18.77	6.52	−12.26	−0.0037	1.23 × 10^−9^
*Escherichia coli*	9.60	15.29	5.69	0.0008	0.0478
*Streptococcus agalactiae*	3.48	2.01	−1.47	−0.0010	0.0013
*Enterococcus faecium*	1.29	2.35	1.06	0.0004	0.0292
*Staphlococcus aureus*	8.05	7.19	−0.86	−0.0014	0.0019
*Staphylococcus haemolyticus*	2.30	1.52	−0.78	−0.0002	0.3783
*Tueperella pyogenes*	1.09	1.53	0.44	1.65 × 10^−5^	0.8542
*Citrobacter koseri*	0.36	0.58	0.22	4.83 × 10^−5^	0.5156

**Table 13:** Statistical significance of increasing and decreasing bacteria–antibiotic combinations observed during the 2022–2024 period.

Microorganism	Antibiotic	Direction	Linear Trend (Coeff.)	*p*-Value
*Streptococcus uberis*	Rifampicin	Decrease	−0.0502	1.81 × 10^−6^
*Streptococcus uberis*	Trimethoprim + Sulfonamides	Decrease	−0.0447	8.10 × 10^−5^
*Streptococcus uberis*	Oxacillin	Decrease	−0.0325	0.0011
*Streptococcus uberis*	Erythromycin	Decrease	−0.0308	0.0025
*Gen. Bacillus*	Trimethoprim + Sulfonamides	Increase	−0.1020	0.0127
*Enterococcus faecium*	Rifampicin	Decrease	−0.0576	0.0135
*Enterococcus faecalis*	Rifampicin	Decrease	−0.0400	0.0304
*Enterococcus faecalis*	Erythromycin	Decrease	−0.0390	0.0391
*Staphylococcus* *haemolyticus*	Trimethoprim + Sulfonamides	Increase	+0.103	0.0367

## Data Availability

All data are subject to privacy protection. If needed, direct access or discussion may be requested by emailing the corresponding author.
